# The gaze bias effect in toddlers: Preliminary evidence for the developmental study of visual decision‐making

**DOI:** 10.1111/desc.12969

**Published:** 2020-04-21

**Authors:** Toshiki Saito, Ryunosuke Sudo, Yuji Takano

**Affiliations:** ^1^ Institute of Development, Aging and Cancer Tohoku University Sendai Japan; ^2^ Japan Society for the Promotion of Science Tokyo Japan; ^3^ Graduate School of Systems Life Sciences Kyushu University Fukuoka Japan; ^4^ Smart‐Ageing Research Center Tohoku University Sendai Japan

**Keywords:** eye movements, gaze bias, preference judgment, toddler

## Abstract

Several studies have investigated the interactive relationship between attention and decision‐making, which is known as the gaze bias effect. Although the generalizability of the gaze bias effect has recently been observed among young and older adults, it remains unknown in which developmental period individuals start to exhibit this relationship. This question was addressed in the current study by recruiting 58 toddlers aged 2–4 years. Participants were asked to do a two‐alternative forced‐choice task in which they chose one of two soft toys they preferred while their eye movements were recorded. Results demonstrated that toddlers exhibited gaze bias regardless of age. We also found that the number of gaze shifts during the task increased according to age. These results suggest that the interactive relationship between attention and decision is acquired by the age of two. The implications of the increased number of gaze shifts for visual decision‐making are discussed.

**Research Highlights**

- We recruited toddlers aged 2–4 years and examined the development of gaze bias in situations where subjects were asked to identify a preferred option.
- We investigated whether gaze bias could be observed in toddlers as young as 2 years.
- The characteristics and time course of gaze bias in toddlers become similar to those of adults as toddlers age.
- The number of gaze shifts observed when participants chose a preferred option also increased with age.

## 1 INTRODUCTION

Preference formation, which is fundamental to personality and daily behavior, is affected by eye movements (i.e. attention). In the last two decades, much research has examined the relationship between decision‐making, such as preference formation, and attention (e.g. Smith & Krajbich, 2018). For example, Krajbich and his colleagues showed that gaze toward an option can be considered to be a facilitator of the option's value and that the value of the option can be modeled computationally by using a measure of gazing time toward the option (Krajbich, Armel, & Rangel, 2010; Krajbich & Rangel, 2011). Moreover, manipulating gazing patterns toward each option unconsciously affects a choice (Pärnamets et al., 2015; Shimojo, Simion, Shimojo, & Scheier, 2003). Thus, it is apparent that attention has a crucial role in decision‐making.

The gaze bias effect is one of the well‐known phenomena demonstrating the role of attention on decision‐making. When people are faced with a decision, their gaze tends to be biased toward the option they finally choose. This phenomenon is known as the gaze bias effect and has received attention from many scholars (Glaholt & Reingold, 2009a, 2009b, 2009c; Mitsuda & Glaholt, 2014; Nittono & Wada, 2009; Saito, Nouchi, Kinjo, & Kawashima, 2017; Schotter, Gerety, & Rayner, 2012; Shimojo et al., 2003).

Although numerous studies have examined the gaze bias effect, there has been less research on its generalizability among different age groups. Most of the studies examining eye movements during a decision‐making task have assessed only the eye movements of young adults, such as undergraduate students and/or graduate students (e.g. Schotter et al., 2012). Thus, scant work has explored the effect of development and/or aging on eye movements during decision‐making. More recently, Saito et al. (2017) tested the generalizability of the gaze bias effect among different age cohorts. They monitored and compared young and older participants’ eye movements during a two‐alternative forced‐choice task in which participants had to choose one of two options. Findings showed that both young and older adults exhibit similar eye movements. Thus, they concluded that the gaze bias effect could be generalized among young and older adults. However, there is no study investigating whether much younger people (e.g. toddlers) exhibit the gaze bias effect.

No studies have investigated whether toddlers exhibit the gaze bias effect. However, many have explored developmental changes in gazing behavior (i.e. visual preference), which is believed to be related to the gaze bias effect. According to these studies, infants tend to prefer familiar stimuli in earlier stages and novel stimuli in later stages (Gollin & Moody, 1973; Houston‐Price & Nakai, 2004). For example, 3‐day‐old neonates showed a visual preference for a familiar face (i.e. their mother's face) over an unfamiliar face when shown both faces simultaneously (Pascalis, Schonen, Morton, Deruelle, & Fabre‐Grenet, 1995). Infants aged 6 to 8 weeks also showed a visual preference for familiar stimuli, whereas 10‐ and 12‐week‐old infants had a visual preference for novel stimuli over familiar stimuli (Wetherford & Cohen, 1973). Novelty preference is reflected in the gazing behavior of adults (Gollin & Moody, 1973). In this respect, the gazing behavior of toddlers reflects visual preferences similar to those of adults from an early developmental stage. Given this similarity in the gazing behavior of toddlers and adults, it is possible that toddlers begin to exhibit gaze bias as soon as they are able to have distinct preferences—even prior to 18 months of age (Valkenburg & Cantor, 2001).

Eye‐tracking methods, especially the preferential looking paradigm, have been frequently used in developmental research. As far as we know, however, few studies have addressed the intentional preference formation of toddlers. The preferential looking paradigm is the most popular technique for assessing implicit cognitive aspects of infants and toddlers who cannot express their thoughts by verbal communication (Fantz, 1964). In an experiment using this paradigm, the experimenter shows participants a number of objects and calculates average looking time at each object. If the looking time differs among objects, the experimenter can infer that they can discriminate the objects. Using this paradigm, many previous studies have shown the extraordinary abilities of infants, such as a preference for prosocial behavior in infants aged 3 months (Hamlin & Wynn, 2011). However, no work has addressed the question of whether infants “prefer” such things. In other words, there is a huge gap between previous studies focusing on preference formation in adults, and that of toddlers and infants.

In this study, we sought to resolve the above two issues in gaze bias research: a dearth of studies of much younger cohorts, and the lack of work in this area comparing adults with toddlers. As discussed above, it is possible that toddlers exhibit the same gaze bias effect that adults demonstrate during decision‐making. To explore this possibility, we examined the eye movements of toddlers aged from 2 through to 4 years. By this period, toddlers have already acquired basic verbal communication ability through general developmental processes (Benedict, 1979; Bloom, 1976).

## 2 METHODS

### 2.1 Participant

We were uncertain of the desired effect size for the present study and determined our sample size by referring to a similar study that demonstrated the generalizability of the gaze bias effect to different age cohorts (Saito et al., 2017). The sample in that study consisted of approximately 20 participants per age group. Therefore, we sought to recruit 20 participants for each of our own groups. Our final sample consisted of 58 toddlers (32 boys, 26 girls), including nineteen 2‐year‐olds (*M*_age_ = 27.37 months, *SD*_age_ = 3.12 months, 6 girls), twenty 3‐year‐olds (*M*_age_ = 39.90 months, *SD*_age_ = 2.90 months, 11 girls), and nineteen 4‐year‐olds (*M*_age_ = 52.05 months, *SD*_age_ = 3.61 months, 9 girls). Participants’ parents received ¥2,000 (approximately $20) for their participation. Participants were recruited through flyers posted at an obstetric clinic and from a database of local families interested in participating in research in the middle southern region of Japan.

### 2.2 Stimuli

The stimuli were 48 soft toys, including 24 toys of familiar characters and 24 toys of unfamiliar characters. We used soft toys because they are easy to grab safely and popular among children. Toys were soft enough that children could grab them easily, and presented no risk of harm if accidentally dropped or bitten by the children. Soft toys are the most popular kind of toy for 2‐year‐olds and the second‐most popular kind of toy for 3‐ and 4‐year‐olds (Rikukawa, 2018). For these reasons, we considered soft toys an appropriate means of engaging children in our task and chose them as stimuli for our study. We used 24 soft toys representing familiar characters from popular cartoons (the Anpanman, SEGA TOYS CO., LTD). We used the familiar toys to motivate children to engage in our task. In Japan, these characters have the highest level of popularity among 2‐ to 4‐year old children (Rikukawa, 2018). However, although we expected that using familiar toys would motivate children to engage in the task, we recognized that the familiarity of the toys could affect the children's behavior. For this reason, we also used 24 unfamiliar soft toys. These toys represented the local mascots of regions of Japan (the Yuru‐kyara, m‐up Inc.). The dimensions of the familiar soft toys were approximately 18 cm × 10 cm × 7 cm. The dimensions of the unfamiliar soft toys were approximately 11 cm × 8 cm × 4 cm. All 48 soft toys were used for every child.

### 2.3 Apparatus

Eye movements were recorded using a camcorder (Sony HDR‐CX420: 60 frames per second). The camcorder was placed between the experimenter and the small box, which was located in front of each participant. The distance between the participant and the camcorder was approximately 60 cm. After the experiment, two researchers coded eye movement using the methodology utilized in a previous study (Shimojo et al., 2003). When a participant looked at the chosen stimulus, a true value of 1 was assigned to every sampling point. When a participant looked at the unchosen stimulus, a false value of 0 was assigned to every sampling point. When a participant did not look at either stimulus, the label “not‐a‐number” was used.

### 2.4 Procedure

A parent of each participant signed an informed consent form. Next, a child and an experimenter faced each other across a small box. First, the experimenter had a brief conversation with the child to make the participant feel comfortable. Then, the experimenter explained the experimental task to the child. We applied a modified version of the two‐alternative forced‐choice task to the present study. The task was chosen for its strong psychometric properties (see Rush, Mortenson, & Birch, 2010, for a review). In this task, the subject is shown a pair of items and asked to pick one. Typically, each item is paired with every other item to allow each to be ranked based on the total number of times it is chosen. However, in the current study, we paired each toy only with one other toy. We did not show toys multiple times because we sought to exclude the effects of repeated exposure on gazing behavior, as was done in a previous study (Shimojo et al., 2003). Details of the task procedure are as follows: First, the experimenter verbally instructed the child to choose the toy that they preferred: “好きな方をとってね” [Please pick a toy that you prefer]. Next, the experimenter used their hands to show two toys to the child. The experimenter also cued the child verbally when the toys were to appear. The verbal prompt used to notify the child of a toy's appearance was “せーの, はい” [Ready? Here]. The distance between the toys and the child was adjusted to be within the child's reach. After the toys appeared, the child chose one of the toys, indicating their preference by grabbing it. While the child made their choice of preferred toy, the experimenter looked down at the floor to prevent the experimenter's gaze from influencing the child's behavior. There were four sessions that contained six trials in the experimental task. Thus, we used all 48 toys (i.e. 24 pairs) with each child. Before the first session, the child performed two training trials. Familiar toys were used in the first trial and unfamiliar toys were used in the second trial. In this way, we ensured that children were familiar with each type of toy and with the nature of the task. Following the training trials, the experimenter told the child that he would show them two soft toys several times—either familiar toys (i.e. Anpanman) or unfamiliar toys (i.e. Yuru‐kyara), similar to those the child had seen in the training trials. He asked the child to choose a preferred toy from a pair of soft toys, as they had performed in the training trials. In each session, the same types of soft toys were used. The order of the toy pairs was manually randomized in each session, but the random numbers method of randomization was not used. The order of sessions was fixed to facilitate the performance of the experiment. The first and third sessions used familiar toys and the second and fourth sessions used unfamiliar toys. In total, each child performed 24 trials in one sitting of approximately 15 min.

This study was approved by the human subject ethics committee of Doshisha University (Number 15,077) and was conducted in accordance with the Declaration of Helsinki.

## 3 RESULTS

### 3.1 Reaction time

To assess the difference in reaction times for choice of stimuli between the three age groups (2–4 years old), we conducted a between‐designed one‐way ANOVA in which age group was an independent variable and reaction time was a dependent variable. We did not find any significant differences among age groups (*F* (2, 55) = 0.656, *p* = .523,*η_p_* ^2^ = 0.023). In addition to ANOVA, we conducted a paired *t* test to assess differences in reaction time for familiar and unfamiliar toy pairs. The results showed that reaction time was longer when choosing between familiar toys than when choosing between unfamiliar toys.

However, we found that familiarity did not have a significant effect on gaze bias and number of gaze shifts (Table 1). Therefore, we combined data for familiar and unfamiliar toys in subsequent analyses.

**TABLE 1:** Comparisons between familiar and unfamiliar stimuli

Variable	Familiar		Unfamiliar		*t*‐value	*p*‐value
	*M*	*SE*	*M*	*SE*		
Reaction time (ms)	2022.741	132.149	2205.572	136.442	2.234	0.029
Gaze bias (ms)	581.654	55.311	785.350	142.108	1.356	0.181
Number of gaze shifts	1.192	0.088	1.270	0.084	1.701	0.094

Note

Degree of freedom was 57.

### 3.2 Gaze bias toward chosen stimuli

**FIGURE 1:**
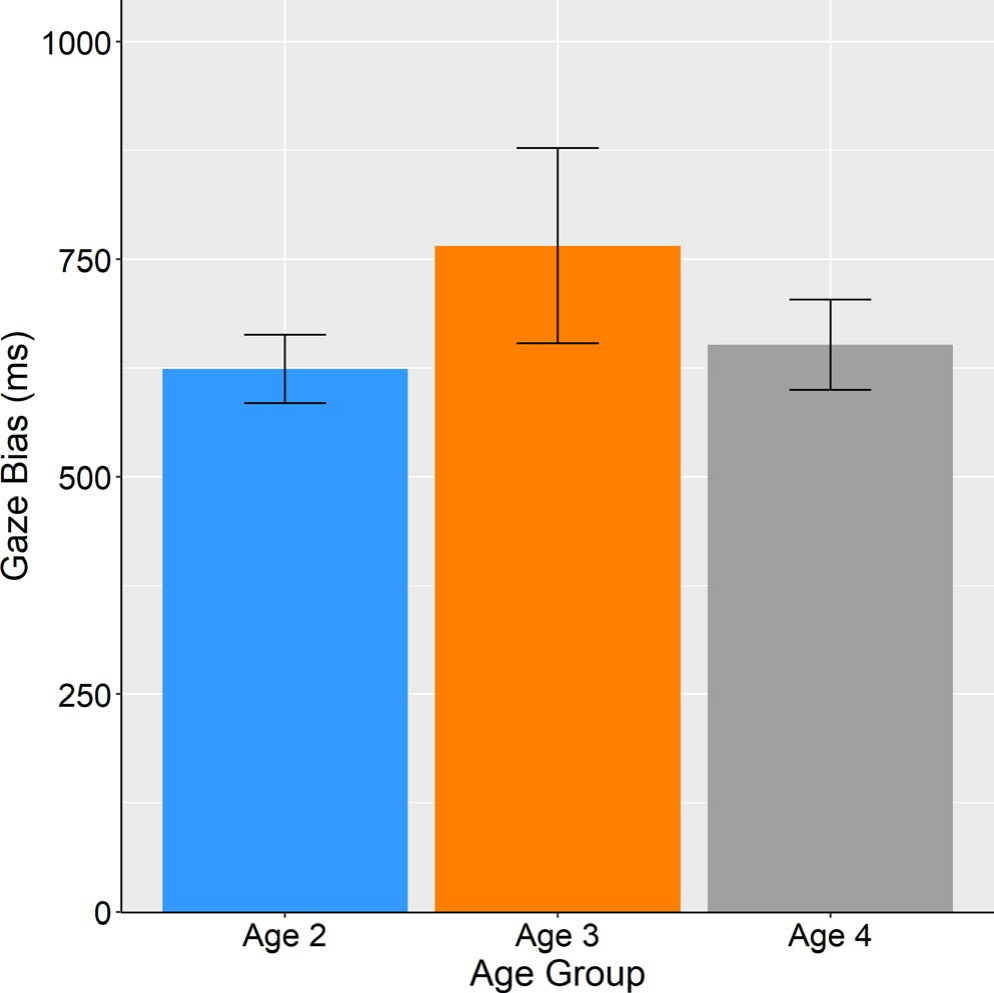
Average gaze bias toward chosen option (total time on chosen option—total time on unchosen option). Error bars represent standard error of the mean

To assess differences of gaze bias toward chosen stimuli among three age groups (2–4 years old), we conducted a between‐designed one‐way ANOVA in which age group was an independent variable, and dwell time on chosen stimulus subtracted from dwell time on unchosen stimulus was a dependent variable (Figure 1). We did not find any significant differences among the age groups (*F* (2, 55) = 0.316, *p* = .731, *η_p_* ^2^ = 0.011). These results suggested that the gaze bias effect is observable even in toddlers aged 2 years.

### 3.3 Gaze likelihood curve

**FIGURE 2:**
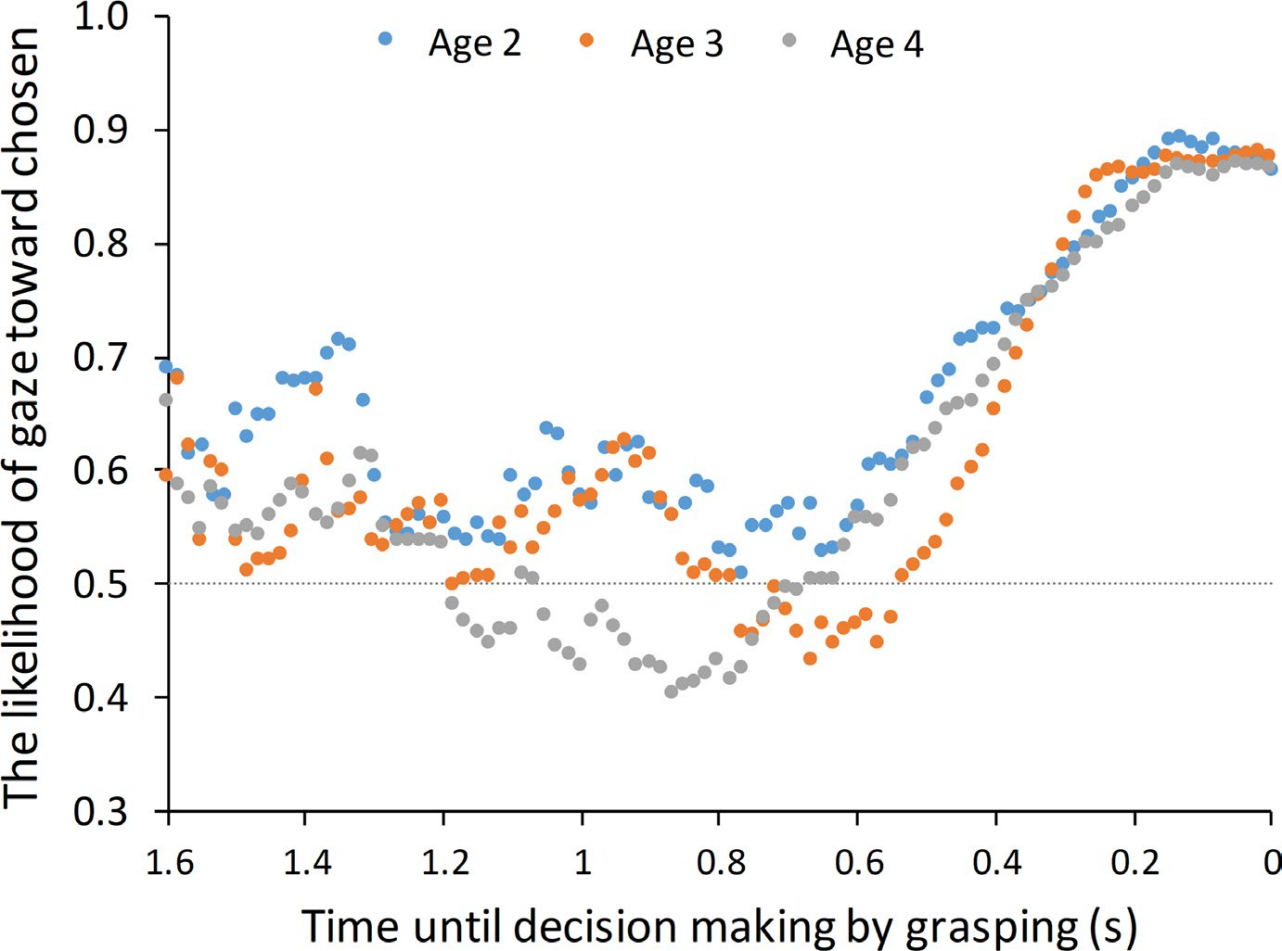
Likelihood of gazing at the chosen option for 1.6 s before grabbing. The horizontal dotted line represents the chance level (50%)

Following previous studies (Saito et al., 2017; Shimojo et al., 2003), we conducted a gaze likelihood curve analysis to analyze the gaze behavior data. The gaze likelihood curve shows the likelihood that the chosen stimuli were inspected at each sampling point. We assigned a true value (1) to every sampling point when a participant looked at the chosen stimulus and a false value (0) when a participant looked at the unchosen one. When participants did not look at either stimulus, we assigned “not‐a‐number.” The gaze likelihood curves represented a progressive bias toward the chosen stimulus in all age groups (Figure 2). Although the heights and the gradient of gaze bias curves did not differ among age groups, the likelihood of the gaze before the start of gaze bias differed among age groups. The gaze likelihood curves of both 3‐ and 4‐year‐old toddlers appeared to drop below 50% before the start of gaze bias. This shape is also observed in previous studies in which adults participated (Nittono & Wada, 2009; Saito et al., 2017; Shimojo et al., 2003). However, the likelihood curve of 2‐year‐old toddlers did not drop below 50% before the start of gaze bias. This difference might represent the developmental process of visual decision‐making. One of the factors causing this difference might be the number of shifts, because the increased number of gazes toward the unchosen option reduced the likelihood gaze. Therefore, we calculated the number of shifts during the decision‐making task and compared this between age groups.

### 3.4 Gaze shift

**FIGURE 3:**
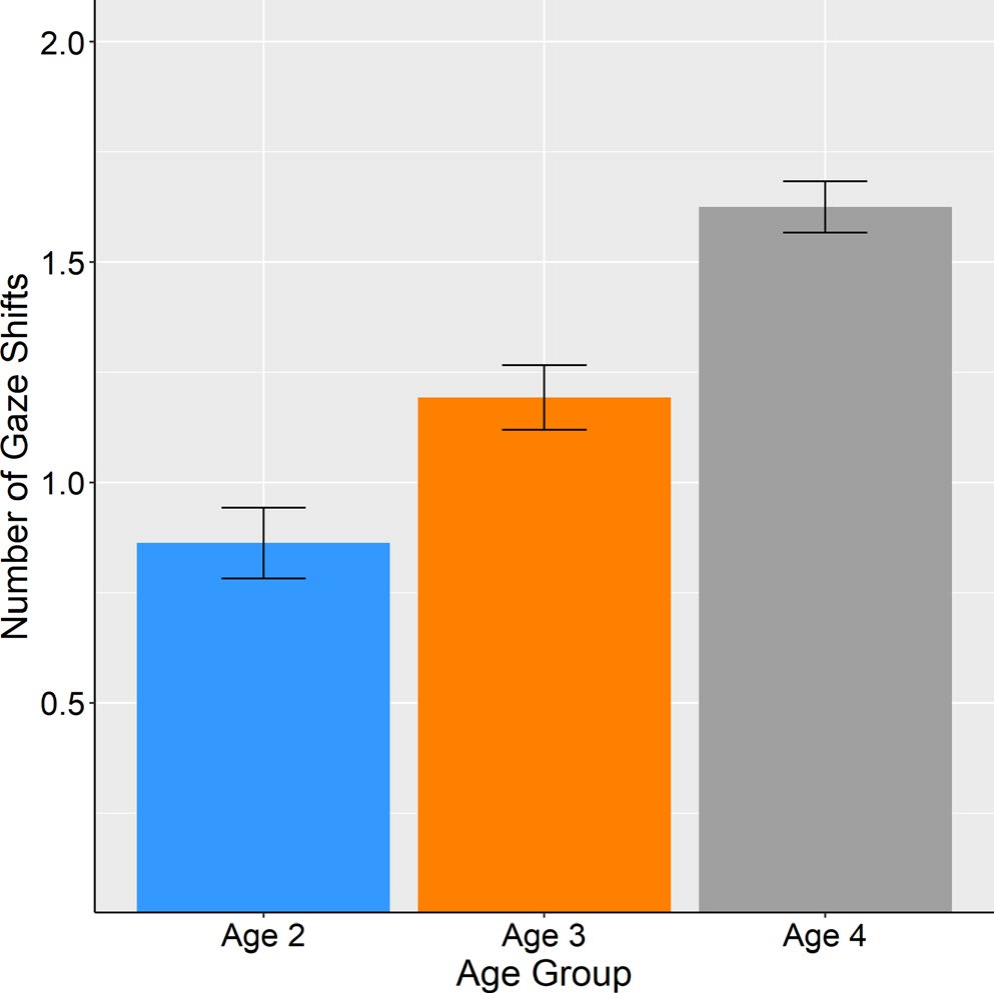
The average number of gaze shifts. Error bars represent standard error of the mean

To assess the effect of age on the number of gaze shifts, we conducted a between‐designed one‐way ANOVA in which age group was the independent variable and the number of gaze shifts was the dependent variable (Figure 3). There was a main effect of age on the number of gaze shifts (*F* (2, 55) = 8.929, *p* < .001, *η_p_* ^2^ = 0.245). Post hoc analysis revealed that 4‐year‐old toddlers shifted their gaze more frequently (*M* = 1.625, *SD* = 0.458) than 3‐year‐old toddlers (*M* = 1.193, *SD* = 0.572, *t* (55) = 2.417, *adj.p* = .019) and 2‐year‐old toddlers (*M* = 0.863, *SD* = 0.630, *t* (55) = 4.213, *adj.p* < .001). There was no significant difference between 3‐year and 2‐year‐old toddlers (*t* (55) = 1.850, *adj.p* = .070, n.s.).

## 4 DISCUSSION

In this study, we sought to investigate in which developmental period individuals start to exhibit the gaze bias effect by conducting a single eye‐tracking study with toddlers. Results showed that toddlers of all age groups gradually biased their gaze toward the stimuli they finally chose, prior to their decision, and that the degree of gaze bias toward the chosen stimuli was not significantly different between each age group. However, the shape of the gaze likelihood curve was different among age groups. The curves of 4‐year‐old toddlers appeared to drop before the start of the gaze bias, with a curve shape similar to that of adults. In contrast, 2‐year‐old toddlers did not manifest this shape. There is a possibility that this different shape was caused by the different number of gaze shifts. Indeed, 2‐year‐old toddlers manifested a significantly lower number of gaze shifts than 4‐year‐old toddlers.

The gaze bias effect we observed in this study implies that the effect is a general phenomenon in toddlers from the age of 2 as well as in adults. In addition to previous studies, which focused on young cohorts such as university students, Saito et al., (2017) showed that the gaze bias effect was also observed in much older cohorts. Consistent with this study, our results also suggest that the relationship between eye movements and decision‐making could be generalized to various age cohorts.

The increased number of gaze shifts with age might relate to the development of deliberative thinking. When the number of gaze shifts is zero, the toddler looked at only one of the two options but not at both options. In contrast, when the number of gaze shifts was greater than zero, the toddler looked at both options before making a decision. The finding that some 2‐year‐old toddlers shifted their gaze less than once (*M* = 0.863) means that the 2‐year‐old toddlers did not engage in a manner that would allow them to determine their preference. In other words, 2‐year‐old toddlers did not consider each option deliberately before making a decision. In contrast, 4‐year‐old toddlers shifted their gaze more than once (*M* = 1.625). Therefore, 4‐year‐old toddlers considered both options deliberately before making a decision. Considering that inhibitory control linearly develops from 2 years to 4 years (Kochanska, Murray, Jacques, Koenig, & Vandegeest, 1996) and that the number of gaze shifts increased likewise in the current study, the number of gaze shifts of 2‐year‐old toddlers might reflect impulsive behavior. In contrast, those of 4‐year‐old toddlers might be related to some developmental process of deliberative thinking.

Findings from the A‐not‐B task are also helpful in interpreting the number of gaze shifts in the current study. In the standard version of the A‐not‐B task, infants watch an object, typically a toy, hidden in one of two locations. After a short delay, infants are allowed to reach for the hidden toy. After a certain number of trials in which the toy is hidden in one location (an “A trial”), the object is hidden in the other location (a “B trial”). A search in location A during a B trial is termed an A‐not‐B error (Marcovitch & Zelazo, 1999). Although the A‐not‐B error gradually decreases with development (Forssman, Bohlin & Hofsten, 2014), performance differs by response modality (i.e., looking versus reaching). Infants performed better on looking trials than on reaching trials between 5 and 7 months old, but performed equally well on both types of trial from 8 to 10 months old (Cuevas & Bell, 2010). Cuevas and Bell (2010) argued that differences in performance, in looking and reaching trials, reflected differences in the maturation of brain circuity associated with the task response. Indeed, looking responses are exhibited at a very early developmental stage (i.e. among neonates), whereas reaching responses are not exhibited until the third or fourth month (e.g. Banks & Salapatek, 1983; Thelen, Corbetta, & Spencer, 1996).

Given that reaching ability develops more slowly than looking ability, increased gaze shifts in the present study might be caused by immature reaching ability and/or incomplete integration of gazing and reaching. In the present study, toddlers had to compare two options and reach for the preferred option simultaneously. Toddlers who had difficulty integrating gazing and reaching might have needed more cognitive resources than more developed toddlers to complete the task. Therefore, it might be the case that 2‐year‐old toddlers lacked the cognitive resources needed to compare two options and showed fewer gaze shifts as a result. Further studies will be needed to clarify the relationship between the number of shifts during decision‐making, the development of cognitive ability, and differences in the maturation of the looking and reaching responses.

This study had several limitations and implications. First, the procedure of the decision‐making task we used in the current study was different from those in previous studies. In most of the previous studies, participants expressed their preference by pressing buttons linked with preferred options (e.g. Saito et al., 2017; Shimojo et al., 2003). However, toddlers expressed their preference by grabbing the preferred options directly. Although this aspect of this study's procedure might have provided more ecological validity than of previous studies, the procedural difference between the current and previous studies might affect eye movements. Thus, further studies in which adult participants express their preference by the same procedure as that of the current study will be needed to ensure the reliability of the results of the current study. Second, we did not ascertain the developmental starting point of the interplay between gaze bias and decision‐making. In the current study, we recruited toddlers over 2 years old because we needed to communicate verbally with them to instruct them in the task. Therefore, to examine the gazing behavior of toddlers much younger than 2 years old, it is necessary to develop an appropriate task where toddlers can express their preferences without understanding verbal instructions. If we could investigate the starting point of the interplay between gaze bias and decision‐making, we would improve our understanding of how our preferences, which are the fundamental to personality and daily behavior, are formed developmentally. Third, we determined our sample size by referring to a similar study (Saito et al., 2017). However, it would have been ideal to determine the sample size based on an estimated effect size by a previous study prior to obtaining data. Thus, a future study with an appropriate sample size based on the results of the present study will strengthen the reliability of our findings. Finally, it was unclear whether the lower number of gaze shifts in 2‐year‐old toddlers was due to failure to engage in the task or limited development of psychological functions related to preference formation. We could not determine whether gaze behavior during the task was genuinely reflective of toddlers’ preferences. We did not evaluate the association between infants’ visual preference and true preference because we chose to model our study procedures after those used in previous studies of the gaze bias effect (Shimojo et al., 2003). To resolve this issue, future studies should use slightly different task procedures. The child participating in the task should be told "Here is X” (experimenter holds one hand forward) and “Here is Y” (experimenter holds the other hand forward). The experimenter should then put both hands out of sight before holding them back up and saying “Pick one.” This would ensure that the child has seen both items and that their visual preference reflects their true preference.

In summary, the results have two implications. First, the gaze bias effect in visual decision‐making would appear to commence in the early stages of cognitive development. Second, decision‐making in toddlers would appear to be deliberative with the development of gaze shift. Therefore, in any future studies concerning visual decision‐making, it is important to consider the role of gaze‐shift development.

## CONFLICT OF INTEREST

T.S., R.S., and Y.T. declare no conflict of interest. The funding sources were not involved in the study design, collection, analysis, interpretation of data, or writing of the manuscript.

## Data Availability

Neither the data nor the materials have been made available in a permanent third‐party archive; requests for the data or materials can be sent to the corresponding author.
